# Diet and diet‐associated bacteria shape early microbiome development in Yellowtail Kingfish (*Seriola lalandi*)

**DOI:** 10.1111/1751-7915.13323

**Published:** 2018-12-01

**Authors:** Jackson Wilkes Walburn, Bernd Wemheuer, Torsten Thomas, Elizabeth Copeland, Wayne O'Connor, Mark Booth, Stewart Fielder, Suhelen Egan

**Affiliations:** ^1^ Centre for Marine Bio‐Innovation School of Biological, Earth and Environmental Sciences University of New South Wales Sydney NSW Australia; ^2^ NSW Department of Primary Industries Port Stephens Fisheries Institute (PSFI) Taylors Beach NSW Australia

## Abstract

The supply of quality juveniles via land‐based larviculture represents a major bottleneck to the growing finfish aquaculture industry. As the microbiome plays a key role in animal health, this study aimed to assess the microbial community associated with early larval development of commercially raised Yellowtail Kingfish (*Seriola lalandi*). We used qPCR and 16S rRNA gene amplicon sequencing to monitor changes in the microbiome associated with the development of *S. lalandi* from larvae to juveniles. We observed an increase in the bacterial load during larval development, which consisted of a small but abundant core microbiota including taxa belonging to the families *Rhodobacteraceae, Lactobacillaceae* and *Vibrionaceae*. The greatest change in the microbiome occurred as larvae moved from a diet of live feeds to formulated pellets, characterized by a transition from *Proteobacteria* to *Firmicutes* as the dominant phylum. A prediction of bacterial gene functions found lipid metabolism and secondary metabolite production were abundant in the early larval stages, with carbohydrate and thiamine metabolism functions increasing in abundance as the larvae age and are fed formulated diets. Together, these results suggest that diet is a major contributor to the early microbiome development of commercially raised *S. lalandi*.

## Introduction

A global increase in consumer demand has resulted in aquaculture being one of the fastest growing sectors in the global economy, with recent estimates that 47% of world fish supplies are now sourced from aquaculture (FAO, 2018). Most fish farming is dependent on the land‐based production of juveniles (larviculture). However, high mortality rates (between 80% and 100% of hatched larvae) in marine fish larviculture systems often result in production bottlenecks (Planas and Cunha, 1999; Lee, 2003; Vadstein *et al*., 2013). While improvements in gamete quality, nutrition and physicochemical conditions in fish rearing systems have made larviculture for some species viable (Merchie *et al*., 1997; Sorgeloos *et al*., 2001), efficiencies remain relatively low (Vadstein *et al*., 2013), suggesting that other factors are important for successful larval rearing.

In recent years, there has been an increased awareness of the importance of the host microbiome for digestion, immune function and development of animals, including fish (Bates *et al*., 2006; Fraune and Bosch, 2010; Stephens *et al*., 2016; Egerton *et al*., 2018; Wang *et al*., 2018). It has been shown that early bacterial colonization of the fish gastrointestinal (GI) tract is caused by the uptake of waterborne (Han *et al*., 2010) and/or feed‐associated bacteria (Hansen and Olafsen, 1999). The fish species’ trophic level and diet preference (Sullam *et al*., 2012; Smith *et al*., 2015; Liu *et al*., 2016; Gajardo *et al*., 2017), as well as variations of environmental factors, such as temperature and salinity, can also influence the types of microorganisms that colonize the gut (Bledsoe *et al*., 2016; Dehler *et al*., 2017).

Yellowtail Kingfish (*Seriola lalandi*) is an economically significant aquaculture species, particularly in the Asia‐Pacific region. While extensive nutritional and physiological research has been carried out on *S. lalandi* (Chen *et al*., 2006; Miegel *et al*., 2010; Bowyer *et al*., 2014), recent attention has turned to the microbial communities associated with this species (Aguilera *et al*., 2013; Ramirez and Romero, 2017; Legrand *et al*., 2018). For example, a culture‐dependent study observed differences in microbial composition of the *S. lalandi* GI tract between juvenile and pre‐adult life history stages (Aguilera *et al*., 2013). More recently, Ramirez and Romero (2017) found that wild‐caught and farmed *S. lalandi* have distinct intestinal microbiomes and suggest this is primarily due to differences in diet availability. While these reports highlight the changing nature of the *S. lalandi* microbiome, nothing is known about the microbiome of this species during early ontogeny in a cultured environment.

**Figure 1:**
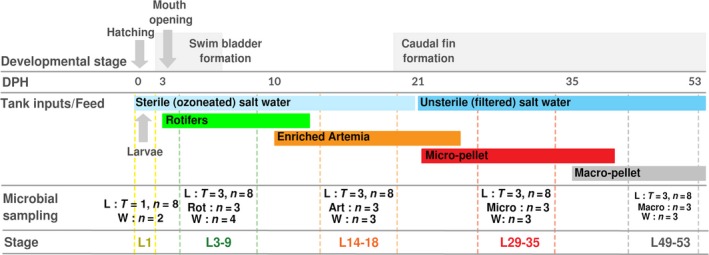
Experimental design and sampling methodology. Key developmental events (top), husbandry events (middle) and sampling procedure (bottom) during the course of the study, on a scale of days post‐hatching (DPH). The number of *S. lalandi* larvae or early juveniles (L), rotifers (Rot), Artemia (Art), micro‐pellet (Micro), macro‐pellet (Macro) and water (W) samples initially sampled at each time point is shown. Note to obtain sufficient biomass for L1 and L3‐9 stage larvae, one sample consisted of pooled individuals, for L14‐18, whole larvae were sampled, and for the juveniles, gut samples were dissected as indicated in the text and are shown in Fig. S1. T = number of time points (where there are more than one), *n* = number of samples per time point.

The aim of this current study was to explore the bacterial community associated with the early developmental stages of *S. lalandi* larvae in a larviculture production facility using 16S rRNA gene amplicon sequencing and functional gene prediction (Fig. 1). As the intestinal microbiome of many species has been shown to be sensitive to host physiology, diet and the environmental conditions, we hypothesized that the *S. lalandi* microbiome varies throughout the early development of the fish. To determine how different sources of environmental bacteria influence the early microbiome development, the waterborne and feed‐associated bacterial communities were also investigated. The findings of this study contribute to a broader understanding of the microbial diversity associated with economically valuable fish species and provide a foundation on which to develop methods for beneficial microbiome manipulation within commercially raised *S. lalandi* larvae.

## Results

### Quantification of bacterial load associated with developing larvae

**Figure 2:**
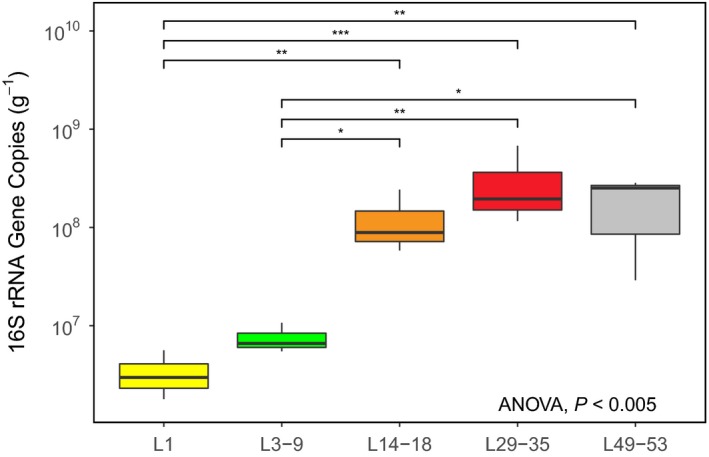
The 16S rRNA gene copy number: Biological triplicates of larvae samples at each life stage (as in Fig. 1) (normalized by the initial mass (g) of starting material. L = larvae/juvenile *S. lalandi*; numbers correspond to the days post‐hatching (DPH). Overall, there was a significant effect of life stage (ANOVA, < 0.005). *Post hoc* pairwise comparisons were performed using Tukey's HSD. The stages L1 and L3‐9, respectively, were significantly different from all later life stages (L14‐18, L29‐35 and L49‐53). Significantly different samples are indicated by asterisk. (*) *** *P* > 0.001; ** *P* < 0.001 > 0.01; * *P* < 0.01 > 0.05.

Quantitative PCR was used to estimate the bacterial load associated with specific development stages of *S. lalandi* larvae and juvenile stages. Bacterial load significantly differed across the larval life stages (one‐way ANOVA, *P* < 0.01, df = 4, Fig. 2), showing a general increasing trend with age. Pairwise comparisons between the larval stages showed a significant increase in bacterial load between the early pre‐feeding (L1 DPH) and rotifer feed (L3‐9 DPH) stages compared to all other later stages (i.e. L14‐18, L29‐35 and L49‐53 DPH).

### Bacterial richness, diversity and bacterial community composition

To characterize the early larval and intestinal microbial community associated with *S. lalandi,* we sampled larvae (L), feed (rotifers (Rot), Artemia (Art), micro‐pellet (Micro) and macro‐pellet [Macro]) and the rearing water (W) at specific stages that coincide with changes to the larval diet (Fig. 1). Sequencing of the V3 and V4 region of the 16S rRNA gene from 131 samples resulted in a total of 8 293 038 sequencing reads after quality filtering and chimera removal. This number was reduced to 5 911 072 reads after the removal of singletons, unclassified sequences, chloroplast and mitochondrial sequences. These sequences were assigned to 6259 OTUs at a 97% sequence similarity cut‐off. OTUs that appeared in less than three samples were removed (~1% of sequences), resulting in a total of 5 847 618 sequences, with a mean read depth of 43 351 sequences per sample, grouped into 2960 OTUs. After rarefaction, three samples (Ma_T1_n1, Ma_T2_n1, Ma_T3_n1) were not sufficiently sampled (< 10 000 sequences) and were thus removed from further analysis. The mean coverage estimate was 0.900288, indicating good sampling of the microbial communities (Fig. S5, Table S1).

**Figure 3:**
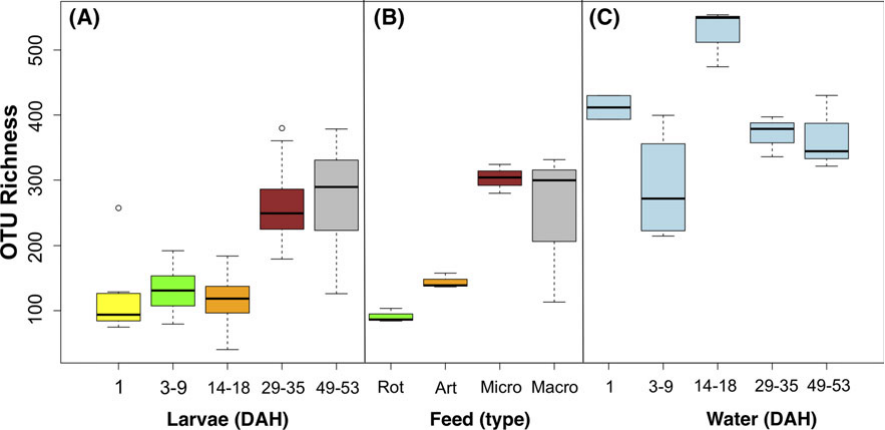
OTU richness: the average number of observed OTUs in (A) the larval microbial community throughout developmental stages, (B) the different feed types, (C) the rearing water during larval development. Colour of larvae (A) corresponds to colour of feed (B) at that stage. Significant differences (assessed by ANOVA with *post hoc* Turkey's HSD) between samples are indicated with an asterisk (*) *** *P* > 0.001; ** *P* < 0.001 > 0.01; * *P* < 0.01 > 0.05.

**Figure 4:**
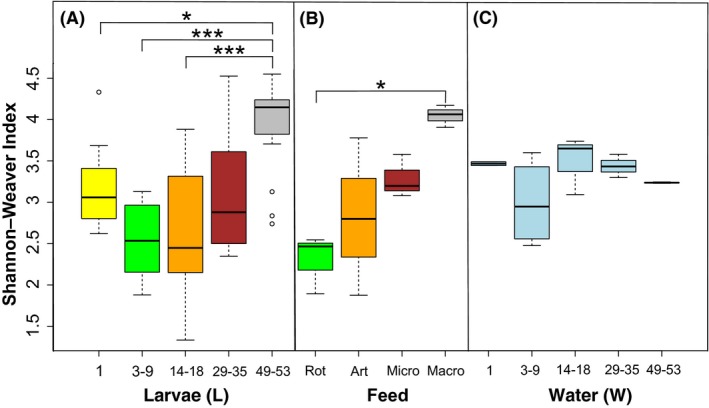
Shannon's diversity: the abundance and evenness of observed OTUs in (A) the larval microbial community throughout developmental stages, (B) the different feed types, (C) the rearing water during larval development. Colour of larvae (A) corresponds to colour of feed (B) at that stage. Significant differences (assessed by ANOVA with *post hoc* Turkey's HSD) between samples are indicated with an asterisk (*) *** *P* > 0.001; ** *P* < 0.001 > 0.01; * *P* < 0.01 > 0.05.

Consistent with the qPCR results, pairwise comparisons between the larval stages showed a significant increase in bacterial richness (number of observed OTUs) between the early (L1, L3‐9, L14‐18 days after hatching (DPH)) and later (L29‐35, L49‐53 DPH) larval stages (one‐way ANOVA, *P *<* *0.001, df = 4, Fig. 3A). Comparisons between the different feed samples (Fig. 3B) also revealed significant differences between the live feeds (rotifers and Artemia) and the micro‐pellet (*P *<* *0.05) and between rotifers and the macro‐pellet (*P *<* *0.001). There was a general increase in diversity with larval age that mirrored the diversity of the corresponding feed samples. Specifically, bacterial diversity (Shannon–Weaver diversity index) was significantly different in the L49‐53 juvenile stage from all other stages (one‐way ANOVA, *P* < 0.001, df = 4, Fig. 4A). Significant differences were also seen between the rotifer and macro‐pellet feed types (one‐way ANOVA, *P* < 0.05, df = 3, Fig. 4B). The bacterial richness (one‐way ANOVA, *P *<* *0.01, df = 4, Fig. 3C) and diversity (one‐way ANOVA, *P *>* *0.05, df = 4, Fig. 4C) of the rearing water were generally high across all stages.

**Figure 5:**
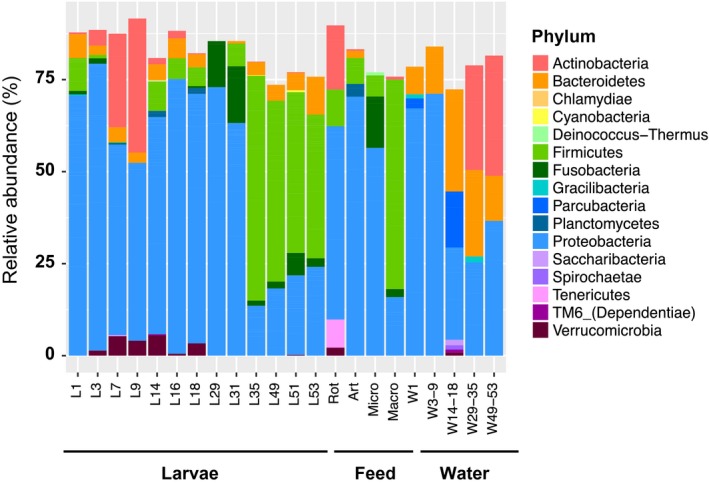
Phylum‐level bacterial taxonomic composition in the *S. lalandi* larviculture system. Relative abundances were calculated using a rarefied library (10 000 sequences per sample), and replicate samples were pooled into sample groups as presented on the *x*‐axis. Relative abundances are only shown for taxa representing > 1% of the rarefied data set sequences.

The bacterial community associated with the larvae, the feed and the rearing water was represented by over 600 genera assigned to 38 phyla. Of the 16 phyla with > 1% relative abundance, four (*Proteobacteria*,* Firmicutes*,* Bacteroidetes* and *Parcubacteria*) comprised 75% of the total OTU abundance (Fig. 5). *Proteobacteria* were more abundant in the early larvae (i.e. from sample L1 to L31); however, from 35 DPH (i.e. sample L35) their relative abundance decreased from > 50% to < 25% and was replaced by *Firmicutes* (up to 61%) (Fig. 5). Other abundant phyla present in the larvae include the *Bacteroidetes* (ranging from 4.5 to 8.0% across all larval samples), *Actinobacteria*, which had an average abundance across all samples of 6%, but showed sharp increases in the L7 and L9 stages (25.8–36.8%), and *Fusobacteria* (3.8% overall), which was enriched in the L29 and L31 stages (to 14.7–15.7%).

In the feeds, *Proteobacteria* dominated the bacterial community of the rotifer, Artemia and micro‐pellet feeds (57.4%, 65.3% and 78% respectively). In contrast, a high abundance of *Firmicutes* (71.5%) was detected for the macro‐pellet. In general, water samples had a high abundance of *Proteobacteria* and *Bacteroidetes*, with the abundance of *Actinobacteria* increasing during the later sampling times (i.e. 27.63% in W29‐35 to 33.83% in W49‐53).

### Variation in bacterial community composition over larval development and identification of the core microbiota

**Figure 6:**
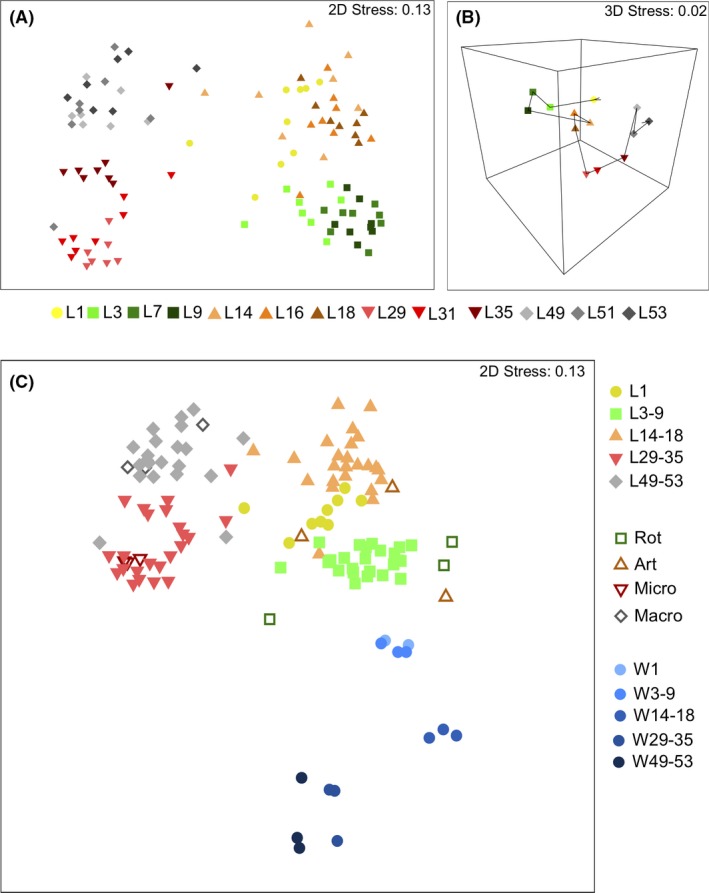
Non‐metric multidimensional scaling (nMDS) plots of: (A) Bray–Curtis distances in 2D of the larval intestinal microbiome across all developmental stages (L1 to L53). Each point represents individual larvae from its respective stage. (B) Bray–Curtis distances in 3D showing the trajectory (as indicated by arrows) of the larval intestinal microbiome over the duration of sampling. Each point represents eight replicate samples grouped into stage categories. Note the direction and length of the trajectory indicate difference. (C) Bray–Curtis distances of all samples in 2D, including larvae and both feed and rearing water. Feed samples are shown with hollow symbols (shape respective of related larval stage), and water samples with blue symbols (circles). Each point represents an individual sample.

Non‐metric multidimensional scaling (nMDS) based on Bray–Curtis dissimilarities of bacterial OTU profiles showed tight clustering of replicates from all larval stages, except for larvae from the L1 stage (PERMANOVA, Pseudo‐*F* = 7.3602, *P *=* *0.001, Fig. 6A). Pairwise comparisons found significant differences between each larval stage (*P *<* *0.05 for each pairwise comparison) with the exception of the comparison between L1 and the L3‐9 stages (*P = *0.09). A trajectory of bacterial composition of the larval microbiome was seen between the larval stages, with the greatest divergence occurring as larvae move from the L14‐18 DPH stage to the L29‐35 DPH stage (*P *=* *0.0002, Fig. 6B).

Specific taxa that were differentially distributed between the larval stages were identified to the family (Table 1) and genus level (Table S5). Once the larvae began to feed, a significant increase in *Rubritaleaceae, Rickettsiales* and *Micrococcaceae* was observed, with the latter taxon decreasing again once the larvae transitioned from a rotifer to an Artemia diet. The greatest changes in bacterial community composition occurred between the L14‐18 and L29‐35 stage when the larvae moved from live feeds to formulated pellets. This included significant changes in 14 families within the phyla *Firmicutes* (including *Lactobacillaceae*,* Clostridiaceae* and *Erysipelotrichaceae*) and a significant increase in a number of *Proteobacteria*, including *Acetobacteraceae, Colwelliaceae* and *Idiomarinaceae*. In contrast, other *Proteobacteria* (e.g. *Rhodobacteraceae, Hyphomicrobiaceae, Legionellaceae* and *Coxiellaceae*), members of *Actinobacteria* and *Rubritaleaceae* significantly decreased in abundance between these two larval stages.

**Table 1:** Summary of differential abundance (log 2) at family level between the larval stages.^a^

Phylum	Family	L1(−) to L3–9 (+)	L3–9(−) to L14–L18(+)	L14–L18 (−) to L29–L35(+)	L29–35(−) to L49–53(+)
*Actinobacteria*	*Micrococcaceae*	6.5469	−6.4190		
	*Microbacteriaceae*	4.8697	−4.1860		
	*Coriobacteriaceae*			7.8825	
	*Corynebacteriaceae*			−2.7468	
	*Mycobacteriaceae*			−6.5874	
	*Propionibacteriaceae*			−3.0615	
*Bacteroidetes*	*Bacteroidaceae*			7.1314	
	*Flavobacteriaceae*			−2.5545	
	*Marinilabiaceae*			7.3638	
*Firmicutes*	*Lachnospiraceae*	−5.3064		6.3081	
	*Acidaminococcaceae*	−5.5100			
	*Veillonellaceae*	−5.0716			
	*Bacillaceae*	−1.8452		1.2327	
	*Acidaminococcaceae*			9.0014	
	*Clostridiales Family XIII*			6.6436	
	*Paenibacillaceae*			6.8693	
	*Lactobacillaceae*			5.0936	
	*Planococcaceae*			5.2877	
	*Peptostreptococcaceae*			4.8280	
	*Clostridiaceae 2*			6.6429	
	*Erysipelotrichaceae*			4.6808	
	*Streptococcaceae*			3.3091	
	*Veillonellaceae*			4.0914	
	*Clostridiaceae_1*			3.8234	
	*Staphylococcaceae*			−1.9404	
	*Thermoanaerobacterales Family III*				3.4843
*Chlamydiae*	*Parachlamydiaceae*			−8.1508	
	*Simkaniaceae*			−6.9379	
*Fusobacteria*	*Fusobacteriaceae*			6.5622	
*Verrucomicrobia*	*Rubritaleaceae*	7.1124		−6.1291	
*Proteobacteria*	*Alcaligenaceae*	−6.0845		6.0186	
	*Rickettsiales*	8.1882			
	*Enterobacteriaceae*	−2.7626	2.0139	−2.0885	
	*Phyllobacteriaceae*		−4.4695		
	*Bacteriovoracaceae*		−6.9515		
	*Colwelliaceae*		−5.9603	6.9977	
	*Hydrogenophilaceae*			5.9285	
	*Xanthomonadaceae*			−2.1733	
	*Thiotrichaceae*			−4.1796	
	*Aeromonadaceae*			−3.3316	
	*Moritellaceae*			5.7658	
	*Campylobacteraceae*			5.2132	
	*Acetobacteraceae*			7.0927	
	*Rhodobacteraceae*			−5.5359	
	*Hyphomicrobiaceae*			−8.6227	
	*Vibrionaceae*			5.0881	−3.1034
	*Legionellaceae*			−6.7005	
	*Coxiellaceae*			−6.6192	
	*Idiomarinaceae*			7.7505	
*Tenericutes*	*Mycoplasmataceae*	8.4540	−9.0815	8.0214	

**a**. Analysis done using DESeq2 package, only differential abundances with *P* < 0.001 are displayed. Note: Negative values indicate a higher abundance in the earlier stage, compared to the later stage, and positive values indicate a higher abundance in the later stage compared to the earlier stage. A log_2_ fold difference is a 2^X^ fold change.

**Figure 7:**
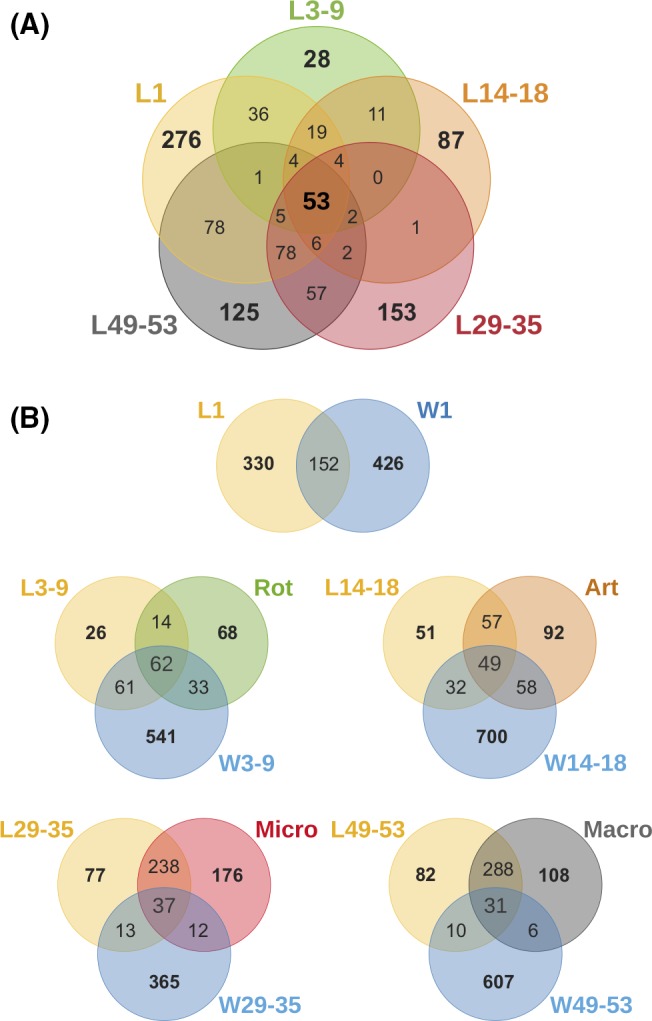
Venn diagrams were constructed exploring rarefied libraries of detected OTUs. Replicate samples were pooled to represent the total number of bacterial phylotypes identified with each sample category. A. displays the extent of shared and exclusive OTUs between *S. lalandi* larvae at their five developmental stages. B. displays the extent of shared and exclusive OTUs between *S. lalandi* larvae or early juveniles (L), the rearing water (W) and the feed (Rot, Art, Micro & Macro).

While significant differences were identified in the larval microbiome between the developmental stages, 53 OTUs (representing 56% of the relative read abundance) were shared across all larval samples regardless of the developmental stage (Fig. 7A, Table S1). These OTUs were therefore identified as potentially core bacteria and included 27 *Proteobacteria* OTUs, 13 *Firmicutes* OTUs, seven *Actinobacteria* OTUs, four *Bacteroidetes* OTUs, one *Cyanobacteria* OTU and one *Deinococcus–Thermus* OTU. Included in the most abundant of these core bacteria are members of the *Donghicola* (OTU2), *Lactobacillus* (OTU28), *Aeromonas* (OTU4) and *Photobacterium* (OTU9) with the relative abundances of each fluctuating across the larval development (Table S1).

### Influence of feed and water samples on larval microbiomes

We also observed significant differences in the microbial community associated with each of the sample types (larvae, feed and rearing water) (PERMANOVA, Pseudo‐*F* = 13.18, *P *=* *0.001, Fig. 6C). With the exception of Artemia, each of the feed samples clustered together with their respective larval stage and these observations are supported by a high coefficient of correlation (Spearman's *r*_s_ = 0.811, *P *=* *0.0001). Similarly, the bacterial community associated with the early stage water samples (W1, W3‐9) showed the greatest resemblance to the community associated with the corresponding larval samples (Spearman's *r*_s_ = 0.691, *P *=* *0.004).

Early stage larvae tended to share a higher number of OTUs with water samples than with the feed (e.g. L3‐9 shared 8.5% OTUs with feed and 37% OTUs with water) (Fig. 7B). This pattern was reversed for the later stages (e.g. L49‐53 shared 70% OTUs with feed and only 2.5% with water). Spearman's rank correlations identified a total of 35 and 22 OTUs from the feed and water samples, respectively, that made a significant contribution to the OTUs present in the larval microbiome (Fig. S6). Of these feed‐associated OTUs, 10 were predominantly assigned to the *Proteobacteria* and were enriched in the early (L3‐9, L14‐18) larval stages, while 11 OTUs, predominantly belonging to the *Firmicutes,* were enriched in the later (L29‐35, L49‐53) larval stages (Table S2, Fig. S6A). The 22 OTUs (Table S3, Fig. S6B) originating from the rearing water showed a more variable pattern, with specific OTUs enriched at different larval stages.

Of the 53 OTUs identified as part of the core larval microbiome, 34 OTUs were found in the initial rearing water and 35 OTUs in the first rotifer feed. From the water, these include OTU11, assigned to *Neptuniibacter* sp., OTU1028 and OTU2, which both belong to the genus *Donghicola*, and are among the most abundant taxa associated with larvae especially in the early stages (Table S1). OTU3 (*Glutamicibacter* sp.), OTU81 (*Vibrio* sp.) and OTU6 (*Nautella* sp.) were among the most dominant of the core larval OTUs also associated with rotifers. Interestingly, the core larval microbiome included five OTUs (OTU205 (*Lactobacillus* sp.), OTU8677 (*Chryseobacterium* sp.), OTU327 (*Psychrobacter* sp.), OTU263 (*Acinetobacter* sp.) and OTU153 (*Corynebacterium* sp.)) that were neither found in the water nor in the first rotifer feed. This suggests that some microbial members appear to originate from the larvae either associated with eggs and/or from environmental contamination, and are maintained throughout development.

### Functional prediction of larval metagenome throughout development

Functional profiles of the microbiome were predicted using Tax4Fun (Aßhauer *et al*., 2015), with approximately 44.4 ± 18.3% of all 16S rRNA sequences being incorporated into the analysis (see Table S4). Statistical analysis revealed that 203 KEGG pathways significantly differed in their abundance across the developmental stages, indicating a major change in the predicted microbiome functionality (Table 2). Differentially abundant pathway function included carbohydrate metabolism, amino acid metabolism, metabolism of cofactors and vitamins, lipid metabolism and metabolism of terpenoids and polyketides (Table 2). Specifically, pathways related to the metabolism of common sugars, including glucose (ko00010), galactose (ko00052) fructose and mannose (ko00051), starch and sucrose (ko00500) were predicted to be more abundant in later stage larvae (L29‐L53). Functions related to thiamine (vitamin B1) metabolism (ko00730) were also more abundant in the later larval stages. In contrast, functions related to lipid metabolism, including unsaturated fatty acids (ko01040), were significantly lower in the older larvae and more abundant in pre‐feeding (L1‐3) or larvae fed on live diets (L3‐L18).

**Table 2:** Mean relative abundances (%) of selected KEGG pathways related to metabolism. Relative abundance of pathways significantly associated with a certain development stage is italicized. Relative abundances were predicted with Tax4Fun

Pathway	Abundance (%)		
	L1	L3‐L18	L29‐L53
Amino acid metabolism	12.359 ± 0.385	12.845 ± 0.707	10.639 ± 0.449
Lysine biosynthesis (ko00300)	0.879 ± 0.035	0.848 ± 0.026	*0.928 ± 0.117*
Lysine degradation (ko00310)	*0.567 ± 0.034*	0.517 ± 0.060	0.455 ± 0.045
Biosynthesis of other secondary metabolites	0.798 ± 0.036	0.813 ± 0.025	0.653 ± 0.088
Penicillin and cephalosporin biosynthesis (ko00311)	*0.166 ± 0.018*	0.160 ± 0.025	0.094 ± 0.026
Streptomycin biosynthesis (ko00521)	0.228 ± 0.009	*0.237 ± 0.011*	0.213 ± 0.017
Carbohydrate metabolism	12.677 ± 0.363	12.442 ± 0.805	13.746 ± 0.502
Glycolysis/Gluconeogenesis (ko00010)	0.883 ± 0.048	0.833 ± 0.045	*1.100 ± 0.157*
Fructose and mannose metabolism (ko00051)	1.204 ± 0.079	1.175 ± 0.204	*1.428 ± 0.126*
Galactose metabolism (ko00052)	0.487 ± 0.083	0.404 ± 0.092	*0.856 ± 0.178*
Starch and sucrose metabolism (ko00500)	1.407 ± 0.141	1.063 ± 0.165	*1.815 ± 0.188*
Lipid metabolism	3.570 ± 0.104	3.653 ± 0.312	3.408 ± 0.188
Biosynthesis of unsaturated fatty acids (ko01040)	*0.257 ± 0.018*	0.256 ± 0.030	0.181 ± 0.035
Metabolism of cofactors and vitamins	7.162 ± 0.131	7.254 ± 0.165	6.862 ± 0.344
Thiamine metabolism (ko00730)	0.574 ± 0.029	0.572 ± 0.035	*0.660 ± 0.072*
Metabolism of terpenoids and polyketides	2.683 ± 0.136	2.536 ± 0.239	2.249 ± 0.335
Tetracycline biosynthesis (ko00253)	0.050 ± 0.003	*0.052 ± 0.006*	0.041 ± 0.006

## Discussion

The host–microbiome of fish plays a key role in growth, development and disease resistance (Kanther and Rawls, 2010; Nayak, 2010; Ran *et al*., 2016; Stephens *et al*., 2016; Egerton *et al*., 2018), yet there is limited information about the diversity and function of these microbes during the course of early development. Here, we examined the microbial community of *S. lalandi* during larval and juvenile development and assessed whether the bacteria from the feed or rearing water act as an inoculum for the *S. lalandi* gut microbiome.

We found the bacterial load of developing larvae significantly increased during the first three stages (i.e. up to L14‐L18), then remained stable. This pattern was reflected in the species richness, showing the increase in bacterial load is likely a result of the introduction of new microbial members and not just the growth of early colonizers. The relatively high levels of bacterial richness and diversity in *S. lalandi* larvae as early as 1 day post‐hatching are also consistent with the early microbiome colonization observed for other commercial fish species ((Califano *et al*., 2017; Li *et al*., 2017) and discussed below). Throughout larval development, only 53 OTUs were shared across all stages; however, these represented 56% of the read abundance, indicating a small, but abundant core community. Species of *Streptococcus, Enterococcus* and *Lactobacillus,* known to be components of the microbiome in healthy fish (Nayak, 2010; Sullam *et al*., 2012; Llewellyn *et al*., 2014) were identified as part of the core microbiome. These genera are typically capable of lactic acid fermentation and may contribute to the stability of the intestinal microbiome in Atlantic cod (*Gadus morhua*), Atlantic salmon (*Salmo salar*) and rainbow trout (*Oncorhynchus mykiss*) (as reviewed in Ringø and Gatesoupe (1998)). Members of the genera *Aeromonas* and *Vibrio* were also part of this core microbiome, but were more abundant during the pre‐feeding (L1) and L14‐18 stages. These bacterial genera are often found in aquatic environments and are known to contain species responsible for some of the most economically important diseases in marine aquaculture and fish production (Toranzo *et al*., 2005). While two sequences related to known pathogens (i.e. *Vibrio tubiashii* (OTU81) and *Vibrio protolyticus* (OTU945)) were detected, most sequences could only be classified to the genus level. Given there were no signs of disease in the larvae, this suggests that these taxa function either as opportunistic pathogens (Brown *et al*., 2012) or represent non‐pathogenic members of the genera (Austin *et al*., 1995).

Outside of the core microbiome, the composition of the bacterial community varied between each stage. Stage‐specific bacterial communities have also been observed for other fish and are thought to result from to a combination of stochastic and deterministic processes (Ingerslev *et al*., 2014; Burns *et al*., 2016; Yan *et al*., 2016; Lokesh *et al*., 2018). With respect to the *S. lalandi* investigated here, the most substantial gut microbiome changes occurred as the larvae transitioned from live feeds to the formulated pellet, suggesting that the temporal variation observed in this study was likely due to the changing diet. However, it is important to note that despite attempts to avoid contamination, these microbiome differences may also be due to a higher abundance of skin or seawater microbes in the younger larval samples, which due to their small size could not be dissected. Nevertheless, other studies have also shown a strong link between fish diet and microbiome membership (Lauzon *et al*., 2010; Gajardo *et al*., 2017; Li *et al*., 2017; Kashinskaya *et al*., 2018). For example, Li *et al*. (2017) observed that the microbiome of the gibel carp (*Carassius auratus gibelio*) was consistent between larvae fed the same diet, and the strongest microbiome shift occurred when the larvae were transitioned from Artemia to a formulated pelleted feed. In contrast, diet and rearing water appeared to have limited impact on the microbiome of cod larvae (*Gadus morhua*) (Bakke *et al*., 2015). Combined, these different observations suggest that both age and the environment can influence microbiome development and the relative influence of either factor may depend on the fish species, reinforcing the importance of system‐ and species‐specific studies.

We found 31% of the total OTUs observed in the early pre‐feeding stage larvae were shared with those in the early stage rearing water (W1; Fig. 7B). This included taxa belonging to *Neptuniibacter, Donghicola* and *Marivita* that are present in the top five most abundant OTUs in pre‐feeding (L1), but in low abundance in older larval and juvenile stages. This pattern has also been observed for channel catfish and other fish species, indicating that the bacterial community in the rearing water has a strong influence at the pre‐feeding stage, while the larvae are still reliant for their development on their endogenous yolk sac. However, in each of these systems the rearing water has less influence once the larvae begin feeding (Bledsoe *et al*., 2016; Califano *et al*., 2017). Interestingly, despite the strong influence of the water microbiota, a large proportion of the pre‐feeding stage OTUs (including five belonging to the core microbiota) appear to have originated from the larvae themselves. While environmental contamination cannot be ruled out, it is plausible that these bacteria represent early egg colonizers that have remained despite ozone sterilization prior to entering the hatchery facility. Indeed, several studies have shown early colonization of newly released eggs (Lauzon *et al*., 2010; Llewellyn *et al*., 2014; Lokesh *et al*., 2018). Future investigation of the microbial community associated with the fertilized eggs and the broodstock environment will be necessary to determine the true source of these early *S. lalandi* microbiome members.

The high community similarity between the diets and the corresponding larval samples indicates direct introduction of the feed‐associated bacteria into the intestine, rather than indirect modulation of the intestinal microbiome by the feed ingredients. The bacterial taxa that became enriched in the early larval stages of the live feed were largely members of the family *Rhodobacteraceae*, which are common marine symbionts involved in both carbon and sulfur biogeochemical cycling (Simon *et al*., 2017). The transition of larvae onto the diet of formulated pellet feeds however, was characterized by a reduction in *Proteobacteria* and an enrichment of *Firmicutes*. These included members of the *Bacillaceae* and *Clostridiaceae*, which are commonly associated with the gut microbiota of animals and known to play an important role in the fermentation of organic matter. *Fusobacteriaceae* were also identified as a main contributor to the gut microbiota from the formulated feed. While this bacterial group is commonly associated with the gastrointestinal tract of freshwater fish, it has also been found in some cultivated marine species (Hennersdorf *et al*., 2016; Tarnecki *et al*., 2017). Although the specific role of *Fusobacteriaceae* for *S. lalandi* is yet to be determined, these bacteria have previously been associated with carnivorous fish (Liu *et al*., 2016; Michl *et al*., 2017), indicating that they may assist in the breakdown of the animal protein components of their diet. Interestingly, the pattern of a shift from a *Proteobacteria* to a *Firmicutes* in the larval microbiota reflects recent observation of the gut microbiome in adult *S. lalandi,* where *Firmicutes* were higher in abundance in aquaculture‐raised kingfish and *Proteobacteria* more abundant in wild‐caught individuals (Ramirez and Romero, 2017). This suggests that the composition of the gut microbiome of aquaculture‐raised animals may be influenced by the introduction of formulated feed, regardless of their age. However, future work will be required to determine both the metabolic activity of these feed‐associated bacteria and their ability to directly colonize the fish intestinal mucosa.

We used Tax4Fun (Aßhauer *et al*., 2015) to predict the gene functions associated with the microbiome during larval development at different major diet changes (i.e. pre‐feeding, live feed and formulated pellets). While confirmation of the functional predictions is required, we observed pathways related to the metabolism of common sugars were more abundant in later stage larvae (L29‐L53). These pathways were similarly enriched in aquaculture‐raised adult kingfish (Ramirez and Romero, 2017) and are likely to be a reflection of the comparatively higher carbohydrate content present in formulated diets than in live feed or wild prey. Of particular note is the high representation of pathways related to the metabolism of starch (ko00500), which is a major carbohydrate constituent of the formulated pellets used here (see reference in Supplementary Material). In contrast, functions related to lipid metabolism were significantly lower in the older larvae and more abundant in pre‐feeding (L1‐3) or larvae fed on live diets (L3‐L18). Interestingly, this includes the gene functions involved in the biosynthesis of unsaturated fatty acids (ko01040), such as the essential omega‐3 fatty acids, that cannot be produced by marine fish larvae, yet are vital for their neural and retinal development (Kristin *et al*., 2013).

Predicted functions involved in amino acid and vitamin metabolism were more abundant in larvae fed on live diets (L3‐L18) possibly reflecting a higher demand for these functions compared to larvae fed on formulated pellets that are generally optimized in terms of micro‐/macro‐nutritional requirements (Kristin *et al*., 2013). However, in some cases deviations from this trend were observed. For example, functions related to thiamine (vitamin B1) metabolism (ko00730) are higher in older larvae (L29‐L53) fed on formulated pellets and may be a result of its role as an essential cofactor in carbohydrate metabolism. In addition, while lysine biosynthesis functions (ko00300) were significantly enriched in older larvae fed on formulated pellets, degradation genes (e.g. ko00310) were generally more abundant in younger larvae during pre‐feeding or live feed stages. Lysine can be a limiting amino acid in many formulated feeds (Mukhtar *et al*., 2017), and the enrichment of functions related to its biosynthesis suggests that GI microorganisms could act as a supplementary source for this important amino acid.

Gene functions related to secondary metabolite production were more prevalent in the early stage larvae. Specifically, the microbiomes of pre‐feeding stage larvae (L1) had a significantly higher proportion of functions related to the synthesis of beta‐lactam antibiotics (ko00311). Functions related to the production of other common antibiotics, including tetracycline (ko00253) and streptomycin (ko00521), were significantly more abundant during the live feeding stages (L3‐L18). These antibiotics are commonly used to treat infections with Gram‐negative bacteria, including several disease‐causing vibriosis (Shaw *et al*., 2014), suggesting that the microbes associated with early larval stages have the potential to produce antibiotics that can protect against pathogenic invaders. However, it is important to note that these are functional predictions only and will need to be confirmed in future studies with more direct measures.

In conclusion, our study contributes to a growing body of work on animal microbiome development. We found that within a commercial larviculture setting, the *S. lalandi* larvae are rapidly colonized by a diverse range of bacteria. Despite a core bacterial community being observed across all larval developmental stages, distinct shifts in the microbiome membership were seen between individual larval stages coinciding with dietary changes. We found shifting to a formulated pellet had the strongest influence on the gut microbiome. During this stage, a high proportion of taxa were shared between the gut and feed samples, suggesting the feed may serve as a continual inoculum for the fish gut microbiome. While the viability of these bacteria is yet to be determined, these results give support to the possibility of supplementing the feed with specific microbial taxa as a means of establishing a functionally beneficial microbiome and improving the performance of reared *S. lalandi* larvae (Merrifield *et al*., 2010; Nayak, 2010). Moreover, the findings here provide an important foundation on which to further investigate the link between the early gut microbiome and the heath of adult *S. lalandi*.

## Experimental procedures

### Rearing of Yellowtail Kingfish (*S. lalandi*), sampling and total bacterial community extraction

This study was conducted under the authorization of NSW Primary Industries (Fisheries) Animal Care and Ethics Committee (ACEC) (REF: 93/1 – Port Stephens). Yellowtail Kingfish (*S. lalandi*) larvae were reared at the NSW Port Stephens Fisheries Institute (PSFI) according to existing commercial production‐scale procedures (Fielder and Heasman, 2011) and further detailed in Supporting information (*See Supporting data file: Rearing of Yellowtail Kingfish* (S. lalandi)).

The sampling scheme for microbial community analysis of the *S. lalandi* intestine aimed to identify the bacteria present at each larval developmental stage that coincides with changing larval feed (Fig. 1). The analysis targeted the bacterial community in (i) the *S. lalandi* larvae, (ii) the feeds (separately: rotifers, Artemia, micro‐ and macro‐pellet) and (iii) the rearing water. Across the developmental timeline, eight independent replicate samples were collected. In addition, three replicate samples of each feed type and a total of 15 rearing‐water samples were collected during larval development (Fig. 1).

*Seriola lalandi* larvae (L) were randomly sampled across the commercial rearing tanks at one time point at the pre‐feeding stage (1 DPH, labelled L1), and samples were then taken at three time points across each of the established developmental stages: rotifer feeding (3–9 DPH, labelled L3‐9), Artemia feeding (14–18 DPH, labelled L14‐18), micro‐pellet feeding (29–35 DPH, labelled L29‐35), macro‐pellet feeding (49–53 DPH, labelled L49‐53) (Fig. 1 and Fig. S1). Samples from L1 and L3‐9 consisted of ~0.05 g (wet weight) larval pools (due to low biomass of individual larvae), corresponding to approximately 10–12 larvae, while samples from L14‐18 consisted of individual larvae. Larval pools and individual larvae were washed with sterile artificial seawater to remove externally attached microbial cells. Samples from L29‐35 and L49‐53 consisted of the individual gastrointestinal tract, as dissection was possible from the juvenile stages. Larval pools, individuals and intestines, were transferred to sterile 1.5 ml polypropylene tubes, snap‐frozen in liquid nitrogen and stored at −20°C. Rearing‐water samples (0.5 l) were sequentially filtered through a 0.45 and 0.22 μm pore‐size filter, with the aid of a 50 ml syringe. Pre‐filtering with 0.45 μm was performed to ensure the removal of any small feed or larval particles. The final 0.22 μm filters were transferred to sterile tubes snap‐frozen in liquid nitrogen and stored at −20°C. Triplicate samples (0.3 g) of each feed type (rotifers, Artemia, micro‐pellet and macro‐pellet) were collected prior to their addition to the tanks. Individual feed samples were snap‐frozen in liquid nitrogen and stored at −20°C until further processing.

Total community DNA was extracted from all samples using the DNeasy^®^ Blood & Tissue Kit (Qiagen, Hilden, Germany) following the manufacturer's instructions. To promote cell lysis before extraction, all samples were initially incubated with 10 mg ml^−1^ lysozyme for 30 min (with vortexing every 15 min) at 37°C (Califano *et al*., 2017) followed by a treatment with 20 mg ml^−1^ proteinase K for 2.5 h at 56°C (with vortexing every 30 min). Extracted DNA was quantified using a NanoDrop (ND‐1000) spectrophotometer (Thermo Fisher Scientific, Waltham, MA, USA).

### 16S rRNA gene copy number determination and analysis

Quantitative PCR amplification of the 16S rRNA gene using the 338F’ (Lane, 1991) and 528R’ (Callens *et al*., 2018) primers was used to determine absolute gene copy number by comparison to a 10‐fold dilution series of the standard fragment (see Fig. S2). The PCR mixture contained 1X PerfeCTa SYBR FastMix (Quantabio, Beverly, MA, USA), 300 nM each primer and 1 μl DNA template with a total volume of 10 μl. The PCR cycling conditions were as follows: activation at 95°C for 5 min, annealing at 40 cycles of 95°C for 15 s and extension at 60°C for 30 s followed by melt curve analysis from 65 to 95°C at 0.5°C intervals. Amplification and analysis were performed using the Mic qPCR Cycler (BioMolecular Systems, Australia) and micpcr software (version 2.6.3). Default settings included using the LinRegPCR method to perform baseline correction and to determine the fluorescence threshold to calculate *C*_q_ and efficiency per sample.

Samples were amplified in biological triplicates with each sample having technical duplicates. Technical duplicates were repeated if they had a standard error >0.5 *C*_t_. The final gene copy numbers per sample were normalized by the amount of biological material extracted. One‐way analysis of variance (ANOVA) tests were performed on log‐transformed copy numbers. *Post hoc* pairwise comparisons using the multcomp package (version 1.4.8) were performed using the Tukey's test to determine significant difference between the groups of interest.

### 16S rRNA gene sequencing and analysis

The V3 and V4 regions of the 16S rRNA gene were amplified from the total community DNA using the 341F’ and 785R’ primers (Klindworth *et al*., 2013). The PCR was performed as described previously (Nielsen *et al*., 2017). PCR products were quantified using gel electrophoresis before being sequenced on the Illumina MiSeq platform with a 2 × 300 bp chemistry at the Ramaciotti Centre for Genomics (UNSW, Sydney, NSW, Australia).

16S rRNA gene reads were processed according to Granzow *et al*. (2017). In brief, sequence data were initially quality‐filtered and trimmed using trimmomatic version 0.36 truncating reads if the quality dropped below 15 in a sliding window of 4 bp (Bolger *et al*., 2014). usearch version 9.2.32 (Edgar, 2010) was used for further processing as described by Wemheuer and Wemheuer (2017) to merge and quality‐filter sequencing reads, excluding reads with < 400 or > 500 nucleotides, in addition to reads with more than one ambiguous base or an expected error of more than 1. Processed reads for all samples were then concatenated and clustered into operational taxonomic units at 97% sequence similarity using the UPARSE algorithm implemented in Usearch. Chimeric sequences were removed *de novo* during clustering and subsequently in reference mode using Uchime with the SILVA database (https://www.arb-silva.de/browser/) (SILVA SSURef 128 NR) as a reference. Remaining OTU sequences were then taxonomically classified by BLASTN (Camacho *et al*., 2009) against the SILVA database with an *E*‐value cut‐off of 10^−20^. All non‐bacterial OTUs were removed along with non‐BLAST aligned and singleton OTUs. Finally, processed sequences were mapped on OTU sequences to calculate the distribution and abundance of each OTU in every sample. Only OTUs occurring in more than two samples were considered for further statistical analysis.

Artificial metagenomes were predicted from the OTU table using Tax4Fun with short read mode disabled (Aßhauer *et al*., 2015). Prior to calculation, OTU sequences were reclassified against the SILVA SSURef 123 NR database with an *E*‐value cut‐off of 10^−20^. Tax4Fun transforms the SILVA‐based OTUs into a taxonomic profile of KEGG organisms, which is normalized by the 16S rRNA copy number (obtained from NCBI genome annotations). Spearman's correlation analysis of functional profiles derived from relevant available metagenome sequences and profiles deduced from 16S rRNA gene sequences revealed a median of the correlation coefficient of 0.76 for the mammalian gut system. This suggests that Tax4Fun provides a good prediction of functional profiles that may be obtained using metagenomic shotgun sequencing approaches. However, it should be noted that predictions are used for hypothesis building and require future confirmation using direct methods.

### Diversity measures and statistical analysis

Measures of community diversity, i.e., OTU richness and Shannon's diversity, were calculated in R (version 3.3.3) using the *vegan* package (Oksanen *et al*., 2014). For the diversity measures, each sample was randomly subsampled (rarefied) to a total of 10 000 counts to account for uneven sequencing depth among the samples. Subsampling was performed 100 times, and the average was taken to reduce randomization effects on our subsampled data as in Nielsen *et al*. (2017). One‐way ANOVA followed by pairwise comparisons using the Tukey's test was performed to determine significant difference between groups. Relative abundances were calculated on the rarefied library (10 000 counts/sample) at the phylum level in R using the *phyloseq* package, and then plotted using the package *ggplot2*. Only taxa that represented > 1% of the rarefied data set sequences were displayed. To identify differentially abundant taxa between the larval stages, the *DESeq2* package was employed considering *P *<* *0.001 (Love *et al*., 2014), with the package presenting the log 2 fold change (2^X^) in bacterial abundance between stages.

For beta‐diversity analysis, the rarefied OTU table was square‐root‐transformed, before measures of community similarity, calculated as Bray–Curtis distances, were created using PRIMER v6 (Anderson *et al*., 2008) and visualized using non‐metric multidimensional scaling (nMDS) (Clarke and Gorley, 2006). Community‐level similarity was compared among larval, feed and water samples using permutational multivariate analysis of variance (Anderson, 2001) with the PERMANOVA add‐on (Anderson *et al*., 2008), which used 9999 permutations of residuals under a reduced model. Samples were further compared using the RELATE function with the Spearman rank correlation method. In addition, BVSTEP analysis was employed to find the smallest subset of OTUs from the feed and water with a multivariate pattern that matched the pattern of the larval community. This involved a between‐samples similarity matrix (Bray–Curtis distances) that used the minimum number of OTUs from the water and feed with a Spearman rank correlation of *P* > 0.90 to the larval OTU similarity matrix. A stepwise procedure with 9999 permutations was used to randomly select subsets of OTUs, with five random starts each (Clarke and Warwick, 1998). The selected OTU subset was removed from the matrix, and the procedure was repeated eight times.

To identify predicted functions and pathways highly associated with development stage of the larvae, a multipattern analysis was applied. For this purpose, the *multipatt* function from the IndicSpecies package was employed (De Caceres and Legendre, 2009). The resulting biserial coefficients (R) of each function/pathway with a particular stage were corrected for unequal sample size using the function *r.g* (Lubomír and Milan, 2006). *P*‐values were subsequently adjusted for multiple testing using the Benjamini–Hochberg correction (Benjamini and Hochberg, 1995).

### Sequence data deposition

Raw 16S rRNA gene sequencing data were deposited in the NCBI Short Read Archive (SRA) under the accession number 454775.

## Conflict of interest

None declared.

## Supporting information


**Table S1**. 53 Core larval OTUs by larval relative abundance.

**Table S2.** Summary of key OTUs identified from the feed that correlate with OTUs in the larvae.

**Table S3**. Summary of key OTUs identified from the water that correlate with OTUs in the larvae.

**Table S4.** Predicted abundances of KEGG pathways and results of the indicator analysis.
